# Etherization in Childbirth

**Published:** 1849-09

**Authors:** 


### Part 2—Reviews and Notices of New Works.

### ARTICLE I.

*A Treatise on Etherization in Childbirth.*—Illustrated by five hundred and eighty one cases—By WALTER CHANNING, M. D., Prof. of Midwifery and Medical Jurisprudence, in the University at Cambridge. Boston 1848. W. D. Tickner, & Co. pp. 400 8vo. (From the publishers.)

This is a valuable work, and one much needed by the profession. Hitherto little has been done with Anæsthetic agents in Midwifery. The denunciations of Prof. Meigs, based we are bound to say, upon insufficient grounds, have deterred many from their use; while others have felt a degree of hesitation in resorting to the use of a powerful class of agents, against which so much has been urged, and whose indiscriminate use is doubtless attended with danger. Mere speculative hypothesis founded on preconceived notions, will never satisfy intelligent and well ordered minds as to the therapeutic vulue of anæsthetic agents: we want facts, and this want is, to a certain extent, supplied by the splendid volume before us. A record of nearly six hundred cases is worth all the theories ever invented, and we heartily subscribe to the quaint aphorism quoted in the title-page, "Give me the facts, said my Lord Judge, your reasonings are the mere guess-work of the imagination." We promise our readers a fuller notice hereafter, and in the mean while recommend them to purchase this work, which, independent, of its professional value, is rendered attractive by the agreeable and scholarly style in which it is written. M

